# T Cells in Gastric Cancer: Friends or Foes

**DOI:** 10.1155/2012/690571

**Published:** 2012-05-31

**Authors:** Amedeo Amedei, Chiara Della Bella, Elena Silvestri, Domenico Prisco, Mario M. D'Elios

**Affiliations:** ^1^Patologia Medica, Dipartimento de Biomedicina, Azienda Ospedaliera Universitaria Careggi, Largo Brambilla 3, 50134 Firenze, Italy; ^2^Department of Internal Medicine, University of Florence, Florence 50134, Italy; ^3^Department of Medical and Surgical Critical Care, University of Florence, Florence 50134, Italy

## Abstract

Gastric cancer is the second cause of cancer-related deaths worldwide. *Helicobacter pylori* is the major risk factor for gastric cancer. As for any type of cancer, T cells are crucial for recognition and elimination of gastric tumor cells. Unfortunately T cells, instead of protecting from the onset of cancer, can contribute to oncogenesis. Herein we review the different types, “friend or foe”, of T-cell response in gastric cancer.

## 1. Introduction

Gastric cancer (GC) is the second cause of cancer worldwide for cancer-related deaths [1]. The regional variations mainly reflect differences in the prevalence of *Helicobacter pylori* infection, which accounts for more than 60% of GC worldwide [2].

*Helicobacter pylori* infection is very common in human populations but only 1% of infected individuals develop gastric cancer in response to persistent infection [2–4]. Certainly *Helicobacter pylori* plays a crucial role [3–5], but also host factors are relevant for the outcome of the infection [6, 7]: actually, many studies showed that the subset of patients who progress to gastric cancer appear to have an increased incidence of some polymorphisms in proinflammatory cytokines and particularly IL-1*β*.

The response of the body to a cancer is not a unique mechanism but has many similarities with inflammation and wound healing. The last century Virchow's [8] observation of a close association between cancer and inflammation anticipated the current interest in the role of immunity in tumor pathogenesis.

Recent insights into the dynamics of the tumor microenvironment have begun to clarify the mechanisms underlying tumor-promoting inflammation, which bears striking similarities to wounds that fail to heal [9, 10]. Approximately 20% of cancer deaths worldwide are currently linked to unresolved infection or inflammation, with gastrointestinal malignancies representing a significant proportion of this disease burden: the most frequent associations are gastric carcinoma and *Helicobacter pylori* infection [3–10], colorectal carcinoma and inflammatory bowel disease [11], pancreatic carcinoma and chronic pancreatitis [12].

Unresolved inflammation generates a microenvironment that facilitates cellular transformation and the propagation of invasive disease. Chronic tissue damage triggers a repair response including the production of growth and survival factors, proangiogenesis cytokines, and immune regulatory networks [7, 8].

The release of inflammatory cell-derived reactive oxygen species coupled with stimulated epithelial cell proliferation creates an elevated risk of mutagenesis. In addition, cross-talk between neoplastic cells and immune elements throughout the smoldering inflammation perpetuates the transforming environment, which provides the evolving tumor cells with sufficient opportunity to acquire mutations and epigenetic alterations that are necessary for cell autonomy.

Inflammatory circuits can considerably differ in different tumors in terms of cellular and cytokine networks and molecular drivers. However, macrophages are a common and fundamental component of cancer promoting inflammation. The drivers of macrophage functional orientation include tumor cells, cancer-associated fibroblasts, B cells, and T cells.

It is not unfrequent that gastric cancer patients with the same TNM stage pursue different clinical courses. Histopathologic classifications, including WHO classification [13] and molecular classifications [14], have also been applied for the prediction of patient survival, but their prognostic accuracies are controversial [13]. In addition, many attempts have been made to link molecular events in cancer cells with patient outcome, but none of these have been proved to be clinically meaningful. As a consequence, new prognostic determinants in conjunction with the TNM stage are required to more reliably and precisely predict patients' clinical course.

As the cancer immunosurveillance hypothesis was first proposed, the concept that the immune system can recognise and eliminate tumor cells has been energetically debated. Many experimental studies in rodents have shown that the immune system indeed functions to protect murine hosts against development of both chemically induced and spontaneous tumors [15]. Furthermore, in humans, epidemiologic investigations indicate that immunocompromised patients have a higher probability to develop cancers of both viral and nonviral origin, which supports the cancer immunosurveillance concept [15]. In addition, current evidence indicates a positive correlation between the presence of lymphocytes in tumor tissue and increased patient survival.

Recent studies have highlighted that several types of tumor infiltrating lymphocytes (TIL) are associated with a better disease outcome for various human cancers [16–18], demonstrating that higher numbers of CD3^+^, CD8^+^, or CD45RO^+^ T cells in tumor tissue are significantly correlated with lower frequencies of lymph node metastasis, disease recurrence, or longer patient survival. Wang et al. advocated that the type, density, and location of immune cells in colorectal cancer have prognostic values that are superior to and independent of those of the TNM classification [16].

However, tumors have developed a number of different strategies to escape immune surveillance, such as the loss of tumor antigen expression, the expression of Fas ligand (Fas-L) or CD200 that can induce apoptosis in activated T cells, the secretion of immunosuppressive cytokines, such as IL-10 or TGF-*β*, or the generation of regulatory T cells, and MHC downregulation or loss [19]. An alteration in HLA class I expression occurs in many cancers, such as gastric cancer [20] and potentially plays a role in the clinical course of the disease by enabling tumor cells to escape T-cell-mediated immune responses [21]. Recent observations suggest that the induction of T-cell apoptosis coexisting with a downregulation of TCR-*ζ* molecules may be responsible for T-cell dysfunction in patients with gastric cancer [22].

Within TIL population, there are also T regulatory cells (Tregs), which are able to inhibit the immune response mediated by CD4^+^ and CD8^+^ T cells in preventing allograft rejection, graft *versus* host disease, and autoimmune disease [23, 24].

In cancer individuals, Tregs were found to downregulate the activity of effector function against tumors, resulting in T-cell dysfunction in cancer-bearing hosts [25, 26]. High numbers of Tregs were indeed reported in patients with different type of cancer [27–29] such as gastric and esophageal cancer [30]. These observations led us to the hypothesis that tumor-bearing hosts with advanced cancers have an increased population of Tregs, which might inhibit the tumor-specific T-cell response.

The aim of this paper is to highlight the role of different T-cell populations involved in gastric cancer immune response and to evaluate their impact in blocking/promoting the development of gastric cancer.

## 2. Protective Role of Cytotoxic T Cells

A large body of evidence indicates that in gastrointestinal malignancies, endogenous responses may inhibit tumor growth and perhaps modulate the clinical course of the disease. Many reports have been obtained on colorectal carcinoma: the type, density, and intratumoral location of the lymphocyte infiltrate have been shown to be more informative biomarkers than the TNM or Duke's classification [16]. In this context, dense infiltrates composed of cytotoxic memory T cells are strongly associated with a reduced risk of recurrence after surgery and increased overall survival. In particular, patients with early-stage cancers but an absence of T-cell infiltrates display poor outcomes, whereas subjects with significant tumor burdens but robust T-cell infiltrates showed improved outcomes [23, 31].

The prognostic role of tumor-infiltrating immune cells in patients with gastric cancer is largely unknown. Only a few reports have been issued on the association between tumor infiltrating immune cells and the clinical outcome in GC: Ishigami et al. [32] reported that patients showing a high level of natural killer cell infiltration in tumor tissues have a better prognosis, and Maehara et al. [33] showed that a high density of dendritic cell infiltration is associated with the absence of lymph node metastasis. On the other hand, the group of Fukuda [34] found no significant difference in survival between patients with marked or slight TIL infiltration. However, they detected TILs by immunostaining in GC patients, classified cases into groups with marked or slight TIL infiltration, and did not determine TIL numbers.

T-cell-mediated adaptive immunity is considered to play a major role in antitumor immunity. In mouse models, it has been demonstrated that adaptive immunity prevents the development of tumors and inhibit tumor progression [35].

Accordingly, recent data [36] showed that in GC high densities of immune cells related to adaptive immunity (especially cytotoxic T cells and memory T cells) are associated with favorable survival and indicate that adaptive immunity plays a role in the prevention of tumor progression.

TIL density is also correlated with the presence of lymph node metastasis but not with the depth of tumor invasion. On the basis of this finding, the authors suspect that the prognostic role of TIL is mainly due to decreased metastatic potential and suggest the following possible mechanisms. First, the expansion of clones with metastatic potential usually containing larger amounts of aberrantly expressed proteins, including proteins that contribute to metastasis, which may act as tumor-associated antigens. As a result, these clones are more likely to be destroyed by in situ immune reactions. Second, a high density of TIL means a healthy immune system, and therefore, immune reaction occurring in lymph node may also exert a proper function against tumor cells that have drained into lymph nodes in patients with high TIL densities. Third, tumor burden of metastatic foci in lymph node is less bulky than those of primary foci, and thus, metastatic foci are more likely to be susceptible to complete destruction by immune reaction.

 Many experimental and clinical observations suggest that metastatic growth in mice and humans is more difficult to control through vaccination and T-cell response, and new observations indicate that immunity against early and even preneoplastic lesions is stronger than against advanced tumors [37–39]. In some cases, enhanced anticancer T-cell activity may thus prevent metastasis rather than eliminating established metastatic nodules. Recently, Kim and coll. [40] evaluated the antitumor activity of *ex vivo *expanded T cells against human GC. For this purpose, human peripheral blood mononuclear cells were cultured with IL-2-containing medium in anti-CD3 antibody-coated flasks for 5 days, followed by incubation in IL-2-containing medium for 9 days. The resulting populations were mostly CD3^+^ T cells (97%): 11% CD4^+^ and 80% CD8^+^. This heterogeneous cell population was also called cytokine-induced killer (CIK) cells. CIK cells strongly produced IFN-*γ*, moderately TNF-*α*, but not IL-2 and IL-4. At an effector-target cell ratio of 30 : 1, CIK cells destroyed 58% of MKN74 human GC cells. In addition, CIK cells at doses of 3 and 10 million cells per mouse inhibited 58% and 78% of MKN74 tumor growth in nude mouse xenograft assays, respectively. This study suggests that CIK cells may be used as an adoptive immunotherapy for gastric cancer patients.

The adoptive immunotherapy of GC with CIK cells has been also reported in preclinical and clinical studies [41]. MHC-I-restricted CTLs from GC patients recognize tumor-associated antigen and react specifically against self-tumor cells [41–43]. One tumor-specific antigen, MG7-antigen, showed great potential for predicting early cancer as well as for inducing immune responses to GC [44, 45]. Using HLA-A-matched allogeneic GC cells to induce tumor-specific CTLs appears to be an alternative immunotherapy option for gastric cancer [38]. Also, CIK cells in combination with chemotherapy showed benefits for patients who suffer from advanced gastric cancers [46, 47]. The serum levels of tumor markers were significantly decreased, the host immune function was increased and the short-term curative effect as well as the quality of life, were improved in patients treated by chemotherapy plus CIK cells compared to those in patients treated by chemotherapy alone [48].

Most studies analyzing T-cell response to tumor-associated antigens (TAAs) have emphasized CD8^+^ T cells thus far. However, CD4^+^ T cells may play a crucial role in both the induction and activation of TAA-specific memory CD8^+^ T cells toward cytotoxic effector T cells [49, 50].

Recently Amedei et al. [51] analyzed the functional properties of the T-cell response to different antigen peptides related to GC in patients with gastric adenocarcinoma. A T-cell response specific to different peptides of GC antigens tested was documented in 17 out of 20 patients. Most of the cancer peptide-specific TILs expressed a T helper 1 (Th1)/T cytotoxic 1 (Tc1) profile and cytotoxic activity against target cells. The effector functions of cancer peptide-specific T cells obtained from the peripheral blood of the same patients were also studied, and the majority of peripheral blood peptide-specific T cells also expressed the Th1/Tc1 functional profile.

In conclusion, in most patients with gastric adenocarcinoma, a specific type 1 T-cell response to GC antigens was detectable and would have the potential of hamper tumor cell growth.

## 3. T Regulatory Cells in Cancer

The physiological role of Tregs is the protection against the autoimmune diseases through the direct suppression of T effector cells reacting against “self,” although they can be also involved in the control of immune response against exogenous antigens [52]. Since most antigens expressed by neoplastic cells are “self”-antigens [53], it is commonly considered that Tregs are also involved in the suppression of the immune response against tumors, favoring tumor escape from immune response [54]. TILs consist of various antitumor effector and regulatory subsets. T-cell infiltration is associated with good tumor prognosis in many types of cancers. CD8^+^ and CD4^+^ T lymphocytes are effector cells thought to be associated with a favorable prognosis [55]. While CD8^+^ T cells are the main effectors of antitumor immunity, CD4^+^ T cells induce and maintain CD8 response [56]. On the other hand, regulatory lymphocytes, a subset of T cells which inhibit the antitumor immune reaction have been described to be associated with unfavorable prognosis [57–62]. Tregs cells are known to attenuate host antitumor immunity by suppressing T-cell proliferation, antigen presentation, and cytokine production [24]. As the tumor progresses and becomes established in the host, the population of TILs is skewed to favor regulatory T cells over the helper CD4^+^ T cells [56].

Studies of regulatory T cells in GC are very few and have yielded conflicting results. Haas et al. [63] reported that stromal but not intraepithelial regulatory T cells are associated with a favorable prognosis. Mizukami et al. [64] reported that the localization pattern but not the absolute number of regulatory T cells was associated with the prognosis. In breast cancer, it has been demonstrated that pathologic complete response to neoadjuvant chemotherapy of breast carcinoma is associated with the disappearance of tumor-infiltrating Foxp3^+^ regulatory T cells [65]. It has been demonstrated that in some kidney tumors Treg frequency is significantly higher in patients with worse prognosis [66]. Conversely, several recent reports highlighted a protective role of Treg in cancer [67]. In renal cancer, Siddiqui et al. showed no correlation between tumor-infiltrating Treg frequency and disease progression [67]. The significance of regulatory T cell in GC as a poor prognostic factor has also been investigated. Perrone et al. [62] and Shen et al. [68] reported unfavorable prognosis with increased intratumoral regulatory T cells.

Two different studies [69, 70] confirmed that GC cell can induce Tregs development via TGF-*β*1 production; in particular the level of serum TGF-*β*1 in GC patients (15.1 ± 5.5 ng/mL) was significantly higher than that of the gender- and age-matched healthy controls (10.3 ± 3.4 ng/mL). Furthermore, the higher TGF-*β*1 level correlated with the increased population of CD4^+^Foxp3^+^ Tregs in advanced GC. A significant higher frequency of CD4^+^Foxp3^+^ Tregs was observed in PBMCs cultured with the supernatant of MGC than GES-1 (10.6%  ± 0.6% *versus* 8.7%  ± 0.7%). Moreover, using the purified CD4^+^CD25^−^ T cells, the authors confirmed that the increased Tregs were mainly induced from the conversation of CD4^+^CD25^−^ naive T cells, and induced Tregs were functional and able to suppress the proliferation of effector T cells. Finally, they demonstrated that GC cells induced the increase in CD4^+^Foxp3^+^ Tregs via TGF-*β*1 production. Gastric cancer cells upregulated the production of TGF-*β*1 and the blockade of TGF-*β*1 partly abrogated Tregs phenotype.

The second study [70] investigated the frequency of Foxp3^+^ Tregs within CD4^+^ cells in TILs, regional lymph nodes, and PBL of GC patients. Furthermore, to elucidate the mechanisms behind Treg accumulation within tumors, authors evaluated the relationship between CCL17 or CCL22 expression and the frequency of Foxp3^+^ Tregs in GC. CD4^+^CD25^+^Foxp3^+^ Tregs were counted by flow cytometry and evaluated by immunohistochemistry. Moreover, an *in vitro* migration assay using Tregs derived from GC was performed in the presence of CCL17 or CCL22. As a result, the frequency of Foxp3^+^ Tregs in TILs was significantly higher than that in normal gastric mucosa (12.4% ± 7.5% *versus* 4.1% ± 5.3%). Importantly, the increase in Tregs in TILs occurred to the same extent in early and advanced disease. Furthermore, the frequency of CCL17^+^ or CCL22^+^ cells among CD14^+^ cells within tumors was significantly higher than that of normal gastric mucosa, and there was a significant correlation between the frequency of CCL171^+^ or CCL22^+^ cells and Foxp3^+^ Tregs in TILs. In addition, the *in vitro* migration assay indicated that Tregs were significantly induced to migrate by CCL17 or CCL22. In conclusion, CCL17 and CCL22 within the tumor are related to the increased population of Foxp3^+^ Tregs, with such an observation occurring in early GC.

Since the Tregs may restrain the antitumor activity of cytotoxic T cells, the balance of effector and suppressor cells may also prove to be a decisive factor in patient outcome, and a recent study [30] contains the first evidence related to the prevalence of Tregs in gastric and esophageal cancer. The authors shown increased populations of CD4^+^/CD25^+^cells in peripheral blood T cells from patients with gastric and esophageal cancers in comparison with healthy donors. Moreover, the population of CD4^+^/CD25^+^ cells in the TILs of GC was higher than that in normal gastric mucosa. Authors also confirmed that CD4^+^/CD25^+^ isolated from patient peripheral blood had a regulatory function by evaluating cytokine production and suppressive activity.

Moreover, the population of CD4^+^/CD25^+^ cells in the TILs of GC patients with advanced disease was significantly more extended than that in TILs of patients with early-stage disease or that in intraepithelial lymphocytes of normal gastric mucosa. As a functional consequence, CD4^+^/CD25^+^ cells did not produce IFN-*γ* but large amounts of IL-10. Also, the proliferation of CD4^+^/CD25^−^ cells was inhibited in the presence of CD4^+^/CD25^+^ cells in a dose-dependent manner, so confirming that CD4^+^/CD25^+^ has an inhibitory activity corresponding to Tregs.

Similar results were obtained by Shen and coll. [71], who demonstrated that increased CD4(+)CD25(+)CD127(low/−) regulatory T cells were also present in the tumor microenvironment, such as those found in the ascites fluid, tumor tissue, or adjacent lymph nodes. In addition, they found that CD4(+)CD25(+)CD127(low/−) Tregs suppressed effector T-cell proliferation and also correlated to advanced stage of GC, suggesting that CD4(+)CD25(+)CD127(low/−) can be used as a selective biomarker to enrich human Treg cells and also to perform functional *in vitro* assays in GC.

## 4. Th17 in Cancer

A new subset of Th cells, named Th17 cells, producing IL-17 alone or in combination with IFN-*γ*, has been identified [72]. Th17 cells may also secrete IL-6, IL-22, and TNF-*α* and play a critical role in protection against microbial challenges, particularly extracellular bacteria and fungi [73]. The role of Th17 cells in tumor immunology can be dichotomous: Th17 cells indeed seem to play a role both in tumorigenesis and eradication of an established tumor. Many laboratories have studied Th17 populations in blood and occasionally tissues of patients with various cancers. A potential protective effect of Th17 cells has been reported in cancer affecting mucosal tissues, such as gut, lung, and skin [74, 75]. An increase in Th17 cells has been detected in peripheral blood, tumor microenvironment and tumor-draining lymph nodes of several different human and mouse tumor types [76], such as ovarian cancer [77].

A recent study has shown that the number of Th17 cells increased in the TILs from melanoma, breast, and colon cancers [78]; Th17 cells were also suggested as a prognostic marker in hepatocellular carcinoma [79]. In contrast to data on solid tumors, little is known about Th17 cells in hematological malignancies. Serum IL-17 levels were recently shown to be elevated in patients with multiple myeloma, especially in stages II and III of the disease. Thus, current data confirm a role for IL-17 in the promotion of angiogenesis and in the progression of multiple myeloma [80]. Th17 cell frequencies and IL-17 concentrations were significantly higher in peripheral blood samples from untreated patients with acute myeloid leukemia than in those from healthy volunteers and were reduced in the former after chemotherapy [81].

On the other hand, some studies have found that the number of Th17 cells is decreased in several types of tumor. The levels of tumor-infiltrating Th17 cells and IL-17 in ascites were reduced in a group of ovarian cancer patients with more advanced disease and seemed to positively predict outcome [82]. A low number of Th17 cell is present in the tumor microenvironment of non-Hodgkin's lymphoma because malignant B cells may upregulate Treg cells and inhibit Th17 cells [83]. Th17 cells are present in much lower numbers in HER2-positive breast cancer patients than in either healthy controls or HER2-negative patients [84].

One study in prostate cancer demonstrated that Th17 cells infiltrating the tumor correlated inversely with the Gleason score [85]. This implied that Th17 cells mediate an antitumor effect in the development of prostate cancer. One group found that IL-17 promoted the tumorigenicity of human cervical tumors in nude mice but inhibited the growth of hematopoietic tumors, mastocytoma P815, and plasmocytoma in immunocompetent mice [86, 87].

It is clear that the Th17 cells have an ambiguous role in cancers: they can both encourage and inhibit cancer progression.

It is well established that IL-17 acts as an angiogenic factor that stimulates the migration and cord formation of vascular endothelial cells *in vitro* and elicits vessel formation *in vivo* [88, 89].

The mechanism of Th17 cells upregulation in tumor is not clear. Charles et al. found that TNF-*α* enhanced tumor growth via the inflammatory cytokine IL-17 in a mouse model of ovarian cancer and in patients with advanced cancer [90]. Su et al. demonstrated that tumor cells and tumor-derived fibroblasts secrete monocyte chemotactic protein 1(MCP-1) and RANTES that mediate the recruitment of Th17 cells [78].

More recently, Kuang et al. showed that tumor-activated monocytes promote expansion of Th17 cells by secreting a set of key proinflammatory cytokines in the peritumoral stroma of hepatocarcinoma tissues [91]. It is clear that Treg cells efficiently suppressed the function of antitumor CD8^+^ T cells [92, 93]. A recent study reported that IL-2 regulates the balance between tumor Treg and Th17 cells by stimulating the differentiation of the former and inhibiting that of the latter in the tumor microenvironment [76].

The mechanism of Th17 cells' antitumor activity remains largely unknown. One recent work has reported antitumor activity of IL-17 by means of a T-cell-dependent mechanism [87].

Two studies by Benatar et al. demonstrated that IL-17E, a cytokine with significant homology to IL-17, has antitumor activity in multiple tumor models, and that eosinophils and B cells are involved in the antitumor mechanism of action of IL-17E [94, 95].

Th17 cells may contribute to protective human tumor immunity by inducing Th1-type chemokines and stimulating CXCL9 and CXCL10 production to recruit effector cells to the tumor microenvironment. A recent study has also demonstrated that almost half of IL-17-producing CD4^+^ T cells isolated from hepatocarcinoma tissues simultaneously produced IFN-*γ* [91].

An interesting work of the Gaudernack group has demonstrated that IL-17-secreting T cell clones obtained from long-term survivors after immunotherapy also secreted IFN-*γ*, IL-4, IL-5, and IL-13 [96]. More recently, it was shown that Th17 cells and IL-17 participate in antitumor immunity by facilitating dendritic cell recruitment into tumor tissues and promoting the activation of tumor-specific CD8^+^ T cells [97].

It was even most intriguing that the Th17 frequencies increased during treatment with trastuzumab in patients with breast cancer [82] or with metastatic melanoma treated with the anticytotoxic T lymphocyte-associated antigen 4 (CTLA4) antibody tremelimumab [10].

Alvarez et al. demonstrated that dendritic and tumor cell fusions transduced with adenovirus encoding CD40L eradicate B-cell lymphoma and induce a Th17 type response in a murine lymphoma model [98]. Moreover, Derhovanessian et al. has observed a highly significant correlation between a higher frequency of IL-17-producing T-cells prevaccination and a shorter time to metastatic progression after immunotherapy [99]. These data imply the important involvement of Th17 cells in the response to cancer immunotherapy (Table 1).

Zhang et al. [100] preliminairly reported that compared with healthy volunteers, patients with GC had a higher proportion of Th17 cells in peripheral blood. The increased prevalence of Th17 cells was associated with clinical stage and in advanced disease increased populations of Th17 cells were present also in tumor-draining lymph nodes. Furthermore, the mRNA expression levels of Th17-related factors (IL-17 and IL-23p19) in tumor tissues and the serum concentrations of IL-17 and IL-23 cytokines were significantly increased in patients with advanced GC. The results indicate that Th17 cells may contribute to GC pathogenesis.

## 5. Concluding Remarks

This paper has highlighted the key roles that T-cell populations play in promotion and/or protection of gastric cancer.

In summary, high densities of cytotoxic T cells and memory T cells are usually associated with favorable survival, indicating the importance of adaptive immunity in the prevention of gastric cancer [41]; as a matter of fact the adoptive immunotherapy of GC with T cells has been also reported in different preclinical and clinical studies [41]. MHC-I restricted CTLs from GC patients recognize tumor-associated antigen and react specifically against self-tumor cells [42, 43], such as MG7-antigen, which shows great potential for predicting early cancer as well as for inducing immune responses to GC [44, 45].

Different studies sometimes reported controversial results, for example, some study showed that Tregs are protective, while others that the Tregs, present in TILs or in peripheral blood of GC patients, are able to suppress the effector T cells, thus promoting the tumor progression [30, 68].

In addition, although not conclusively, recent data suggested that Th17 cells might somehow contribute to GC pathogenesis [96].

On the basis of clinical and experimental evidence, it is reasonable to conclude that the T immune response in GC has double faces as Janus, one friend and one foe, and that to obtain successful immunotherapy might involve a combined approach, which intensify the effector functions of cytotoxic T cells and probably reduce the suppressive T cells.

## Figures and Tables

**Table 1:** Th17 cells in cancer.

Type of cancer	Organism	Role	Effect of subject	Reference
Pancreatic cancer	Mouse	Antitumor	Slower tumor growth and increased survival	Gnerlich et al. [101]
Melanoma	Mouse	Antitumor	Increase in activated CD8^+^ T cells and better antitumor efficacy	Sharma et al. [102]
Melanoma	Mouse	Antitumor	Th17-polarized cells were better at tumor eradication than Th1-polarized cells	Muranski et al. [103]
Ovarian cancer	Mouse	Pro-tumor	Lead to myeloid cell recruitment in the tumor environment and accelerated tumor growth	Charles et al. [90]
Hepatocellular carcinoma	Mouse	Pro-tumor	Decrease of intratumoral Th17 was associated with decreased tumor growth	Kuang et al. [91]
Prostate cancer	Human	Pro-tumor	Higher pretreatment Th17 numbers correlated with faster disease progression	Derhovanessian et al. [99]
Ovarian cancer	Human	Antitumor	Th17 levels correlated positively with patients survival	Kryczek et al. [82]
Prostate cancer	Human	Antitumor	More highly differentiated Th17 in prostate correlated with slower disease progression	Sfanos et al. [85]
Lung adenocarcinoma	Human	Antitumor	Th17 accumulation correlated positively with patient survival	Ye et al. [104]
